# Multivariate Models of Adult Pacific Salmon Returns

**DOI:** 10.1371/journal.pone.0054134

**Published:** 2013-01-11

**Authors:** Brian J. Burke, William T. Peterson, Brian R. Beckman, Cheryl Morgan, Elizabeth A. Daly, Marisa Litz

**Affiliations:** 1 National Marine Fisheries Service, National Oceanic and Atmospheric Administration, Northwest Fisheries Science Center, Fish Ecology Division, Seattle, Washington, United States of America; 2 National Marine Fisheries Service, National Oceanic and Atmospheric Administration, Northwest Fisheries Science Center, Fish Ecology Division, Newport, Oregon, United States of America; 3 National Marine Fisheries Service, National Oceanic and Atmospheric Administration, Northwest Fisheries Science Center, Resource Enhancement and Utilization Division, Seattle, Washington, United States of America; 4 Oregon State University, Cooperative Institute for Marine Resources Studies, Newport, Oregon, United States of America; Technical University of Denmark, Denmark

## Abstract

Most modeling and statistical approaches encourage simplicity, yet ecological processes are often complex, as they are influenced by numerous dynamic environmental and biological factors. Pacific salmon abundance has been highly variable over the last few decades and most forecasting models have proven inadequate, primarily because of a lack of understanding of the processes affecting variability in survival. Better methods and data for predicting the abundance of returning adults are therefore required to effectively manage the species. We combined 31 distinct indicators of the marine environment collected over an 11-year period into a multivariate analysis to summarize and predict adult spring Chinook salmon returns to the Columbia River in 2012. In addition to forecasts, this tool quantifies the strength of the relationship between various ecological indicators and salmon returns, allowing interpretation of ecosystem processes. The relative importance of indicators varied, but a few trends emerged. Adult returns of spring Chinook salmon were best described using indicators of bottom-up ecological processes such as composition and abundance of zooplankton and fish prey as well as measures of individual fish, such as growth and condition. Local indicators of temperature or coastal upwelling did not contribute as much as large-scale indicators of temperature variability, matching the spatial scale over which salmon spend the majority of their ocean residence. Results suggest that effective management of Pacific salmon requires multiple types of data and that no single indicator can represent the complex early-ocean ecology of salmon.

## Introduction

The adult spring run of Chinook salmon (*Oncorhynchus tshawytscha*) in the Columbia River, U.S.A. is comprised mostly of hatchery fish [1], but also includes wild fish from Evolutionarily Significant Units (ESUs, which are the basic management unit for Pacific salmonids) listed under the Endangered Species Act [2]. After spending a year in freshwater, these fish migrate downstream and spend one to five years in the ocean, though the majority come back to the Columbia River after two years [3]. Recent research has shown that juvenile salmon survival in the first few months after leaving freshwater is one of the largest determinants of cohort size [4]–[7]. Although size-selective mortality occurs at least through the first ocean year [8], [9], specific mechanisms of mortality are not well described, making estimates of the number of fish returning to the river elusive. Harvest of adults is divided among Native American ceremonial and subsistence, recreational, and commercial fisheries [10]. The harvest allocation and schedule incorporates a sliding scale, dependent on the total run size of upriver spring Chinook salmon. Therefore, a pre-season forecast of the number of adult fish returning to the Columbia River is required each year to determine harvest quotas for the various user groups.

Successful recovery and conservation of these threatened and endangered salmon, while maintaining the availability of unlisted fish for harvest, requires a good understanding of biological, chemical, physical and hydrological dynamics, each of which can greatly influence population dynamics [11]. These processes are often driven by a wide array of biotic and abiotic variables, resulting in complex interactions between a species and its environment. Yet common statistical and modeling approaches encourage relatively simple designs [12] that often limit the number of predictor variables. As a result, these complex ecological dynamics are often modeled with a relatively simple set of predictor variables [13]–[15]. Moreover, in many ecological studies, limits on both data availability and mechanistic understanding can lead to the use of model covariates somewhat removed from the specific ecosystem processes involved.

Research and management groups currently make pre-season predictions of adult salmon returns using methods with varying degrees of complexity and accuracy. These include moving averages [14], generalized additive models [15], [16], spawner-recruit relationships [17], time series analysis [18], [19], and neural networks [20]. One of the simplest and most common methods involves a sibling regression model, which uses the abundance of returning precocious males (i.e., for spring Chinook, these are fish that spend only one winter in the ocean, often referred to as jacks) as an indicator of adult returns. Such models are based on a correlation between jack counts in one year and adult counts the following year [13], [21]. These sibling models have a variable degree of accuracy, mainly due to their reliance on a stable age structure in salmon populations [10], an assumption that does not always hold [3]. Furthermore, these models require waiting until the year prior to the adult return year before making a prediction. If the marine environment is a large driver of cohort size, indicators of ocean conditions during the year juvenile salmon migrate from the river ought to be useful in predicting adult returns 2 and 3 years later, which would provide managers the ability to generate multi-year planning scenarios.

In recent years, data representing various aspects and processes of the marine ecosystem have been collected and are proving to show strong relationships with salmon survival [22]–[24]. Although encouraging, this presents a dilemma for researchers: how does one incorporate newly-available, often multi-faceted data into analyses that have traditionally favored simplicity? Complicating the situation is the fact that many existing predictor variables exist in long time series’, whereas many of the promising new indicators of the marine environment only go back a decade or less [24].

**Figure 1:**
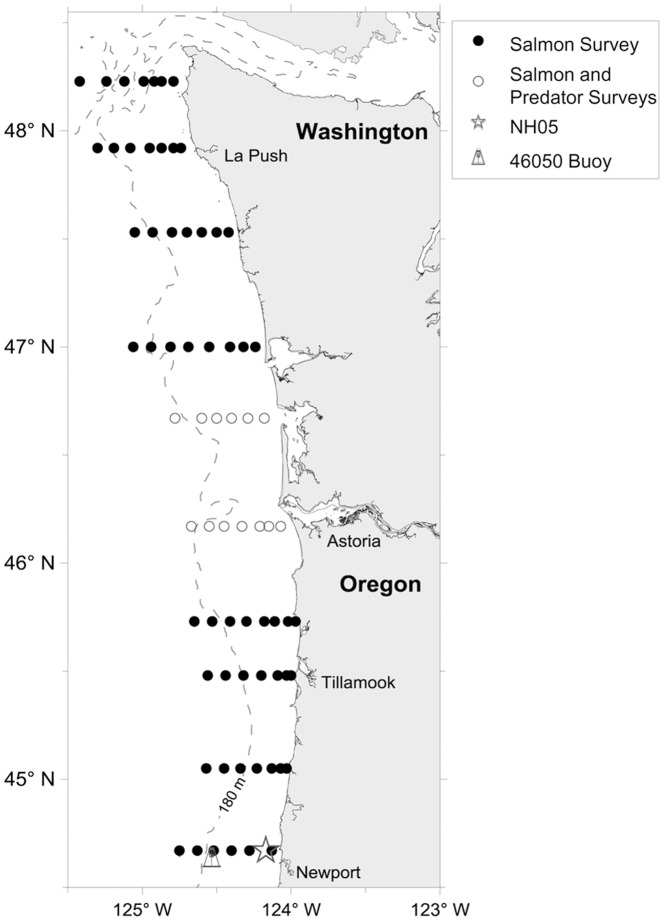
Map of the study region. Sampling locations are shown for the Salmon and Predator Surveys, the NH05 site, and Buoy 46050.

Given the vast area and high cost of sampling the coastal environment, determining direct, causative factors of marine mortality through experimentation was impractical. Since 2000, we have surveyed the coastal environment in an attempt to better understand the physical and biological processes that relate to early marine survival of Pacific salmonids [24] (Figure 1). Through this effort, we identified multiple correlates, or “indicators,” of salmon survival. However, the variance in salmon returns explained by each of these metrics differs significantly. Moreover, as each indicator represents part of an ecosystem with multiple complex interactions, many of these metrics covary (i.e., they are not independent) and this multicollinearity violates many of the assumptions in most statistical procedures. We therefore needed methods to summarize indicators of the marine environment and examine how they relate to salmon returns.

In an effort to collate diverse and complex information into a single management tool, researchers at NOAA Fisheries and Oregon State University used 18 marine indices during the juvenile migration year in essentially a qualitative manner to estimate salmon returns (http://www.nwfsc.noaa.gov/oceanconditions) [24]. This approach has two main benefits: 1) it avoids the pitfall of relying too heavily on one or two covariates and 2) it allows prediction two years in advance. However, there are some aspects of this work that could be improved upon.

First, the covariates, or indicators, included in the above analysis represent a restricted subset of potential indicators, using measures of the Pacific Decadal Oscillation (PDO), the Oceanic Niño Index (ONI), temperature and salinity of coastal waters, coastal upwelling, copepod community structure, and the catches of juvenile Chinook and coho salmon in surveys conducted during their first summer at sea. However, we know that many other ecological processes, such as predator and forage fish abundance [25], prey availability [22], [26], [27], and physiological condition and ontogeny [28], [29], are important to salmon growth and survival during their first ocean year, and should be useful in forecasting. These are not used in the Peterson et al. [24] approach because the time series are not as long as the ones used in the qualitative forecasting (the 18 indicators used by Peterson et al. [24] are compiled from 1998 through 2011, whereas many other indicators only go back to 2000). Second, the non-parametric “mean rank” method gives equal weight to all of the indicators, and therefore does not take advantage of the unequal predictive power of the various indicators, nor does it address the multicollinearity among indicators.

In this paper, we employ a multivariate statistical technique that can a) incorporate a large number of potential indicators, b) give higher weights to indicators that are more related to salmon returns, and c) appropriately handle the multicollinearity among indicators. Our goal was to determine the best combination of indicators to explain the abundance of spring Chinook salmon returning to the Columbia River each year. The multivariate techniques we used resulted in two important products: a pre-season forecast of adult salmon returns, primarily for management of the fisheries, and a measure of indicator importance, which can improve understanding of ocean ecology and guide future marine research. Moreover, the pre-season estimates obtained through these analyses can be used as a starting point for more detailed in-season management adjustments [30], [31].

## Methods

### Data

We collated 31 indicators that represent some aspect of the physical or biological conditions in the marine environment (Table 1). We tried to encompass many types of indicators varying in spatial extent from large portions of the North Pacific Ocean (e.g., the Pacific Decadal Oscillation (PDO) and Oceanic Niño Index (ONI)) to local summaries of biological information (e.g., copepod species richness off the coast of Newport, OR). Temporal coverage varied from biweekly research cruise data, to indicators computed from monthly data (PDO, ONI, upwelling), to intermittent summer research cruises (Figure 1). We assembled data for yearling Chinook salmon smolt out-migration years 2000–2010 (using a 2-year lag, this represents return years 2002 through 2012).

**Table 1:** Name, category, and description of all indicators used in the analysis.

Indicator	Description
	**Category 1– Large-Scale Oceanic and Atmospheric (N = 3)**
PDO.Dec.Mar	Standardized values for the PDO index, derived as the leading PC of monthly SST anomalies in the North Pacific Ocean, poleward of 20°N. Values are summed from December the previous year through March of the ocean entry year, http://jisao.washington.edu/pdo/PDO.latest
PDO.May.Sep	Standardized values for the PDO index, derived as the leading PC of monthly SST anomalies in the North Pacific Ocean, poleward of 20°N. Values are summed from May through September during the ocean entry year, http://jisao.washington.edu/pdo/PDO.latest
ONI.Jan.Jun	Anomaly from the Nino 3.4 region, averaged from January through June of the ocean entry year, http://www.cpc.ncep.noaa.gov/data/indices/
	**Category 2– Local and Regional Physical (N = 10)**
SST.Buoy46050	Annual anomalies of sea surface temperatures (SST) from Buoy 46050: Stonewall Banks –20 NM west of Newport, OR (Figure 1)
SST.Nov.Mar	Average seasonal SST from biweekly cruises off of Newport at NH05 from November the previous year through March of the ocean entry year (Figure 1)
SST.May.Sep	Average seasonal SST from biweekly cruises off of Newport at NH05 from May through September of the ocean entry year (Figure 1)
PhysTransition	The date on which deep water colder than 8°C was observed at the mid shelf (station NH05, Figure 1)
UpwellingAnomaly	A measure of upwelling anomalies for 45°N 125°W averaged from April through May of the ocean entry year, http://www.pfel.noaa.gov/products/PFEL/modeled/indices/upwelling/NA/data_download.html
UpwellSeasonLength	Same data as above, but indicates the elapsed time between the begin and end of the upwelling season, estimated from the cumulative upwelling index following Bograd et al. [54]
DeepTemp	Mean temperature at 50-m depth at station NH 05 (Figure 1, average water depth 60 m) averaged over all biweekly cruises from May to September of the ocean entry year
DeepSalinity	Mean salinity at the 50-m depth at station NH 05 (Figure 1) averaged over all biweekly cruises from May to September of the ocean entry year
DARTFlow	Average daily flow at Bonneville Dam during April and May of the ocean entry year, http://www.cbr.washington.edu/dart/river.html
DARTTemp	Average daily temperature at Bonneville Dam during April and May of the ocean entry year, http://www.cbr.washington.edu/dart/river.html
	**Category 3– Growth/Feeding (N = 13)**
CopRichness	Average number of copepod species in a plankton sample averaged from May through September of the ocean entry year at NH05 (Figure 1), for further detail on the relationships between copepod species richness and oceanographic conditions, see Hooff and Peterson [55]
NCopAnomaly	Biomass anomaly of northern species of copepods, May through September of the ocean entry year
NH05CCI	Copepod Community Index (CCI), copepod community composition Non-metric Multidimensional Scaling (NMDS) x-axis scores of copepod community composition from biweekly surveys at Newport line (NH05; Figure 1), from Keister et al. [56]
BioTransition	Day of year when a northern (cold–water) copepod community first appeared at station NH 05 (Figure 1). We call this this “biological spring transition”
IchthyoBiomass	Average winter ichthyoplankton biomass (mg C×1000 m^−3^) from the Newport Line biweekly surveys (Figure 1), January through March of the ocean entry year, restricted to the top five items in salmon diet
IchthyoCI	Winter ichthyoplankton species community ordination score from an NMDS, January through March of the ocean entry year, restricted to the top five items in salmon diet
MayChDiet	May Chinook salmon diet species community [22]. These are ordination scores from an NMDS analysis on species composition – the particular direction of the association with salmon returns is therefore arbitrary
JuneChDiet	June Chinook salmon diet species community [22]. These are ordination scores from an NMDS analysis on species composition – the particular direction of the association with salmon returns is therefore arbitrary
MayChCond	Length-weight residuals, based on all yearling Chinook caught in May during the Salmon Survey
JuneChIGF	Average insulin-like growth factor (IGF) from yearling Mid- and Upper Columbia River spring Chinook salmon caught in the Salmon Survey
JuneCCI.BPA	Copepod Community Index (CCI). Consists of vertical net copepod community composition NMDS x-axis score from all June Salmon Survey stations
JunBongoBiomass*	Average biomass in Salmon Survey bongo net hauls, restricted to potential prey items for juvenile salmonids
Age1Anchovy*	Age-1 anchovy density (No./km towed <125 mm FL) caught in May and June during the Predator Survey the year following salmon ocean entry (these fish represent the survivors of the cohort that would have been salmon prey size (30–80 mm) during the ocean entry year [57] [No cruises were done in May or June 2010, value for this year was estimated as the mean of all other years]
	**Category 4– Predation/Disease (N = 2)**
AdultHake*	Adult hake density (No./km towed >300 mm SL) caught during the Predator Survey [25]
RsalCh*	*Renibacterium salmoninarum* prevalence in yearling Chinook salmon May through June, obtained from samples collected during the Salmon Survey
	**Category 5– Cohort Abundance (N = 3)**
JunChCatch*	Average catch of Chinook salmon in the June Salmon Survey (fish/km)
CanChCatch*	Catch per unit effort for juvenile Chinook salmon off the west coast of Vancouver Island in June and July (median value of the bootstrap distribution) [52]
ChJacks*	Number of spring Chinook salmon jacks (precocious males) counted at Bonneville Dam the year prior to adult returns (same cohort as response variable, lagged by 1 year), http://www.cbr.washington.edu/dart/river.html

* variables were natural-log transformed prior to analysis.

**Figure 2:**
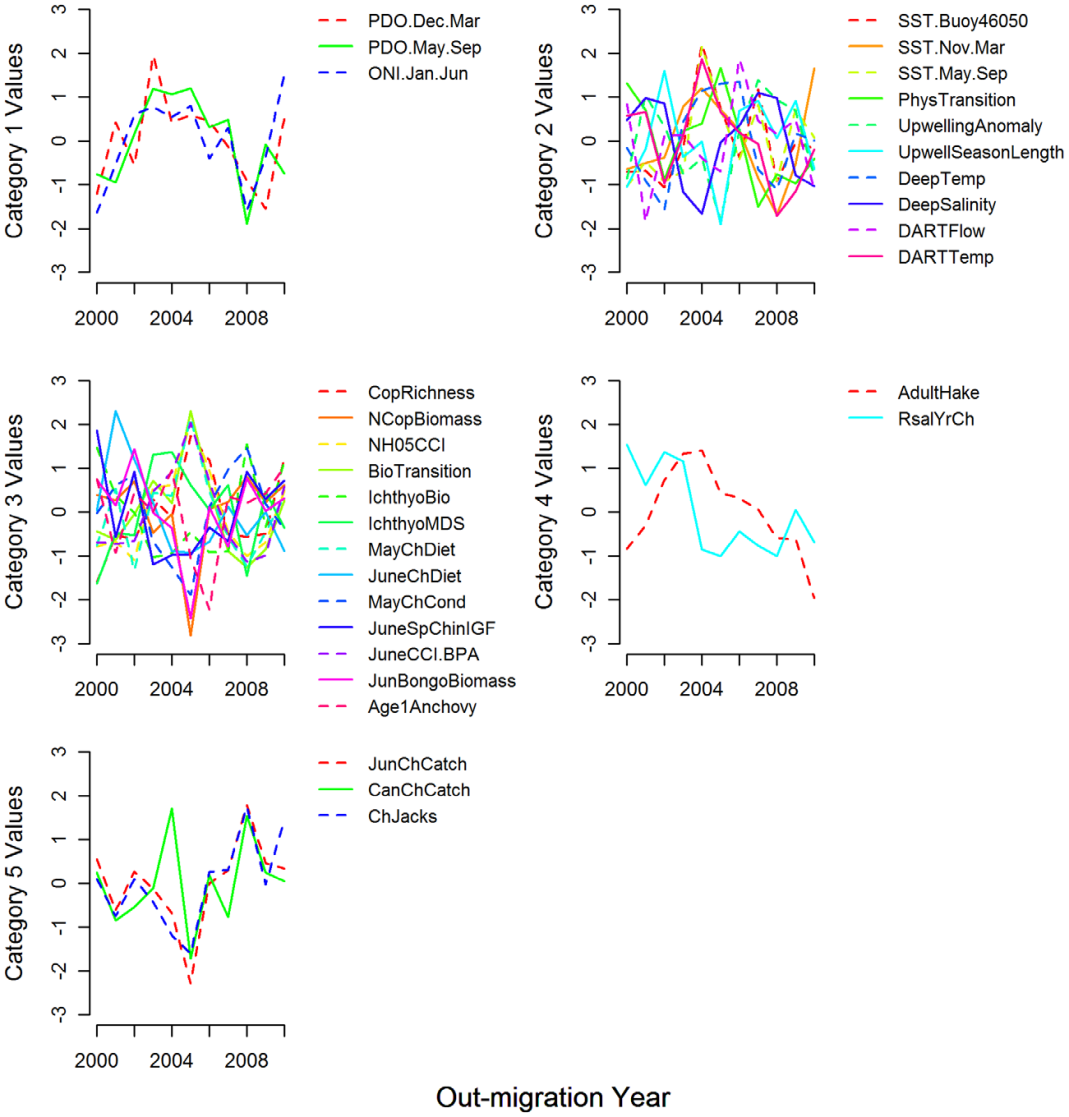
Time series of the 31 indicators, organized by category. All indicator data were scaled to have a mean of 0 and a standard deviation of 1. Indicator categories include 1) large-scale oceanic and atmospheric, 2) local and regional physical, 3) growth/feeding, 4) predation/disease, and 5) cohort abundance.

We sorted the indicators into five categories based on spatial extent and mechanistic relationships with salmon (Table 1, Figure 2). Category 1 includes the large-scale oceanic and atmospheric variables such as the PDO and the ONI. Category 2 contains ten indicators that represent more local or regional variables such as sea surface temperature (SST.Buoy46050, SST.Nov.Mar, and SST.May.Sep), upwelling (PhysTransition, UpwellingAnomaly, and UpwellSeasonLength), or deep water conditions (DeepTemp and DeepSalinity). Two of the Category 2 indicators (DARTFlow and DARTTemp) characterize information from the Columbia River (representing the environment that salmon inhabited just prior to migrating into the ocean). Category 3 (13 indicators) represents ecosystem processes or attributes related to growth and feeding, such as copepod metrics (CopRichness, NCopAnomaly, NH05CCI, BioTransition, and June CCI.BPA), ichthyoplankton (IchthyoBiomass and IchthyoCI), and salmon diet and condition (MayChDiet, JuneChDiet, MayChCond, JuneChIGF, Age1Anchovy, and JunBongoBiomass). Only two indicators (AdultHake and RsalCh) are in Category 4 (representing predation and disease), exemplifying the lack of data on salmon predators. Finally, Category 5 contains three indicators of cohort abundance (JunChCatch, CanChCatch, and ChJacks). These metrics are counts of siblings (i.e., from the same cohort as the response variable). We point out the distinction here between jacks (precocious adult males, ages 1–2), which were part of the predictor data set, and adult Chinook salmon (age 3–5), which was the response variable.

Each indicator was chosen specifically for its potential mechanistic relationship to salmon survival. Indicators were used to describe returns of spring Chinook salmon to specific ESUs (see below). Many indicator data sets were collected via our coastal salmon surveys, which have been conducted almost every May, June, and September since 1998 (Figure 1; see Peterson et al. [32], Brodeur et al. [33] for details on this survey; data were collected under Endangered Species Act Section 10 permit #1410-7A). Other indicator data came from various coastal surveys, and details regarding these sampling efforts can be found in Emmett et al. [25]. To maximize performance in multivariate analyses, we performed an initial check for normality for each indicator, natural-log transformed those indicators with a non-normal distribution (Table 1), and scaled all indicator data to have a mean of zero and standard deviation of one.

In separate analyses, we modeled three response variables representing different portions of the spring Chinook salmon run (Table 2). The first was the annual return of adult spring Chinook salmon, which represents the counts of fish at Bonneville Dam (the first dam on the Columbia River that salmon must pass during their return migration to spawn) through June 15^th^ plus the estimated number of fish harvested in the lower river [10]. Ideally, we would have modeled marine survival (smolt to adult return rates), as we believe most of our marine indicators relate most directly to survival, but the lack of good estimates of smolt abundance precluded this. However, using adult returns as the response variable has direct management implications, as pre-season harvest levels and dates are set based on forecasts of this quantity.

**Table 2:** Chinook salmon adult return data.

Juvenile migration Year	Adjusted Counts at Bonneville Dam	Counts at Ice Harbor Dam	Counts at Priest Rapids Dam
2000	335,214	111,814	34,066
2001	242,605	99,044	17,441
2002	221,675	89,970	12,890
2003	106,911	36,866	14,148
2004	132,583	33,974	8,535
2005	86,247	36,063	6,708
2006	178,629	76,809	11,784
2007	169,296	79,291	13,469
2008	315,345	130,771	30,539
2009	221,157	96,064	15,246
2010	203,063	86,139	19,495
Mean	201,157	79,710	16,756

Spring Chinook salmon counts at Bonneville Dam from Jan 1^st^ through Jun 15^th^ adjusted by estimated lower river harvest (wdfw.wa.gov/fishing/cre/staff_reports.html), counts at Ice Harbor Dam from Jan 1^st^ through Aug 11^th^, and counts at Priest Rapids Dam from Apr 15^th^ through Jun 13^th^ (www.cbr.washington.edu/dart/adultruns.html). All Chinook salmon counts were natural-log transformed for analysis.

The other two response variables approximate returns of specific adult Chinook salmon ESUs. The first was adult salmon counts at Priest Rapids Dam, which encompass the endangered Upper Columbia River spring-run Chinook salmon ESU, and the second was adult counts at Ice Harbor Dam, which encompass the threatened Snake River spring/summer-run Chinook salmon ESU. These latter two response variables were subsets of the first, as fish counted at Priest Rapids Dam and Ice Harbor Dams also contribute to the Bonneville Dam count. We included these ESU-related response variables to show how different stock groups are modeled with different variable weighting, and also to allow between-ESU comparisons, which can be ecologically informative. For example, some indicators used in the analyses may be more appropriate for one ESU or the other, and the multivariate approach described here can help tease this apart. Data from all three Chinook salmon response variables were natural-log transformed prior to analysis.

### Statistics

As adult return data were not available for the 2010 out-migration year, we used data from the 2000 through 2009 out-migration years for model fitting. With 10 years of adult salmon return data and 31 indicators, multiple regression was not an appropriate tool. Even if there were only a few indicators, their potential multicollinearity would present difficulties for a typical regression analysis. To optimally and appropriately use the collective information in the indicator data set, we used two multivariate statistical methods to relate the indicator data to the salmon return data: principal component regression (PCR) and maximum covariance analysis (MCA). After extensive testing on simulated data with known response variables, PCR and MCA were chosen from a longer list of potential multivariate methods, including stepwise selection of indicators and partial least squares regression, because they performed at least as well as the others but had fewer complications and relied on fewer assumptions.

The first step in PCR is to perform principle component analysis (PCA) on the indicator variables. The objective of PCA is to summarize the variance (or structure) in a dataset with as few dimensions as possible by taking linear combinations of the original indicators, which are known as principal components (PCs) [34]. For each PC, the coefficients of the indicators are known as the PC’s “loadings”. For these data, PCA was appropriate because it can represent almost all of the variance in the indicators in a small number of new variables. Another important feature of PCA is that the resulting PCs are orthogonal, which eliminated the problem of multicollinearity in a regression using the original indicators.

In a procedure known as principle component regression (PCR), w used the PCs obtained from PCA as predictor variables in a linear regression analysis (PCR) of adult salmon returns [35]. Because PCR maximizes variance in the indicator data set without regard to relationships with the response variable (i.e., adult salmon returns), it is possible that the first few PCs obtained from a PCA, although representing the greatest amount of variation in the indicator matrix, are not the best predictors of salmon returns. One option in this case is to use backwards stepwise elimination of PCs, keeping only those that contribute significantly to the regression [36]. However, there is a trade-off between keeping more PCs, which improves the model fit, and over-fitting. To remain conservative in model fitting, we used a backwards stepwise selection process on the PCs using Akaike’s Information Criterion corrected for small sample size (AICc) to determine which subset of PCs fit the data in the most parsimonious way [36]. We also considered only the first five PCs as potential independent variables in the PCR, which represented over 88% of the variance in the original 31 indicators.

The second method, MCA, is similar to PCR except that it first calculates the covariance matrix between the indicators and the response, and then runs a PCR on the covariance matrix (as opposed to the indicator matrix). For any single response vector (i.e., a particular salmon population), MCA provided only one principal component. Therefore, there was no need for AICc selection of PCs, and a simple linear regression was performed between the lone PC and salmon returns. This analysis is mathematically identical to calculating a weighted average indicator vector using the covariance values as weights. In this sense, it is directly comparable to, yet an improvement upon, the mean rank analysis currently used [24].

To determine model performance for PCR, we calculated the fitted R^2^ of the model. However, it is inappropriate to use the R^2^ from a fitted MCA model as a measure of model performance because MCA uses information from the response variable in the model (via the covariance matrix). We therefore ran a complete leave-one-out cross-validation for both the PCR and MCA models. From this, we sequentially removed each year, recalculated the PCs and reran the regressions, and calculated the root mean squared error of prediction (RMSEP) to use for model comparison and performance [37].

To address which indicators, or sets of indicators, best explain adult spring Chinook salmon returns to the Columbia River, we quantified the relative contribution to the regression of each of the indicators [34]. Specifically, we multiplied the squared loadings from the PCA (since the squared loadings sum to the eigenvalue, this represents the indicator-specific proportion of overall variance accounted for by each PC) by the semi-partial correlation coefficient for each PC (i.e., the correlation between each PC and the response variable). When summed across PCs (i.e., for each indicator), this provided the total amount of variance in the response variable that was explained by each indicator. We applied the same procedure for MCA, but it was simplified somewhat because there was only one PC.

As many of the indicators are similar in spatial/temporal scale and some have a similar ecological interpretation, we averaged the indicator importance values by category. We used the indicator importance from MCA in this summary for two reasons. First, loadings obtained from PCR can be sensitive to inclusion/exclusion of particular indicators. In contrast, the loadings obtained from MCA, which are directly related to the covariance between each indicator and the response variable, are less likely to shift around in future analyses. Second, variable importance values from MCA were specific to the response variable used (because the loadings were informed by the response), which allowed us to compare the relative importance of indicators across response variables.

## Results

**Figure 3:**
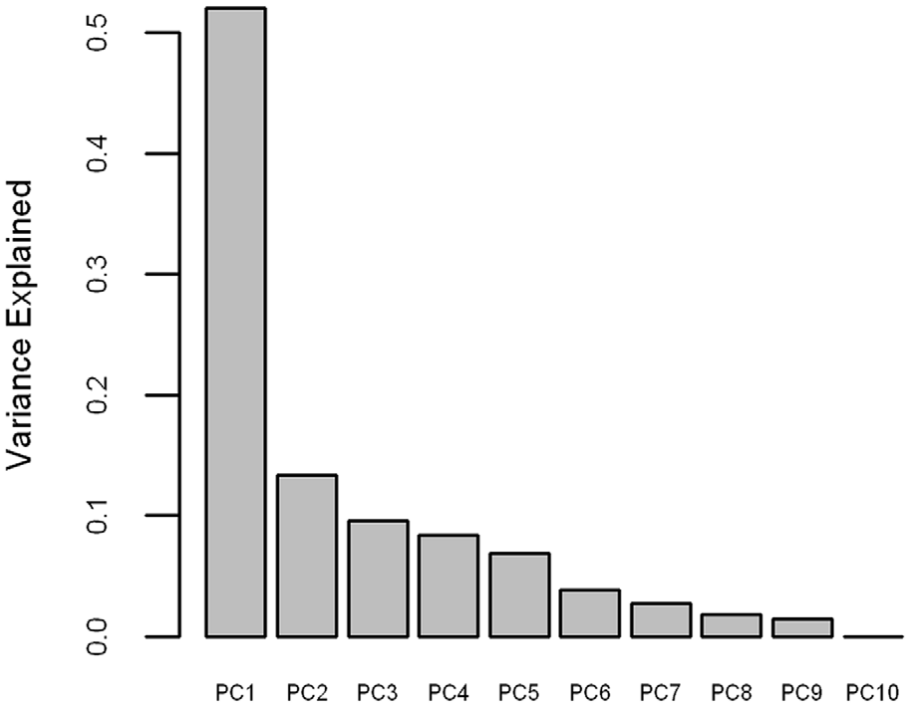
Proportion of variance explained. Proportion of variance in the original indicator dataset explained in by each principal component.

There was a high degree of multicollinearity within the indicator data set, which resulted in an efficient reduction of dimensions using PCA. Statistically, only the first PC was significant (determined through a Monte Carlo randomization test [38], not shown), accounting for over 52% of the variance in the original indicator space (Figure 3). Comparison of model fit using AICc also suggested that only PC1 should remain in the models. It should be noted, however, that some of the less significant PCs were also correlated with the salmon return data (with just 10 data points, AICc penalized the model greatly for each additional parameter). Although all 31 indicators contributed to PC1, there was more than an order of magnitude difference among the relative contributions, exemplifying the power of this analysis over taking a simple average of the indicators.

**Figure 4:**
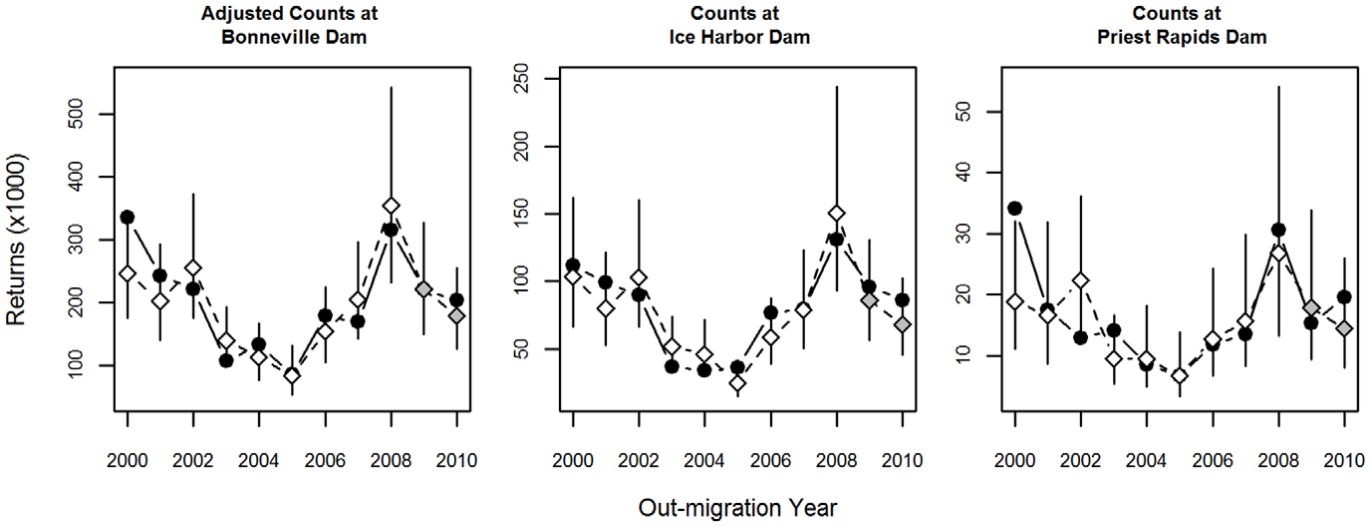
Observed and predicted spring Chinook adult returns. Observed spring Chinook adult returns (solid circles) and leave-one-out predictions (open diamonds) with 95% prediction intervals obtained from MCA. Predicted returns in 2011 and 2012 (2009 and 2010 juvenile migration year) are shown with 95% prediction intervals (grey diamonds).

Model fits were strikingly similar between PCR and MCA (Table 3), despite the different weighting schemes used by these two methods. Predictions, 95% prediction intervals, and RMSEPs were almost identical between PCR and MCA. We scaled the RMSEP to the average observed returns so we could compare relative model performance across response variables. The models performed much better for the two response variables of greater magnitude (larger returns); the scaled RMSEPs from both PCR and MCA were 0.2 for spring Chinook salmon entering the mouth of the Columbia River and 0.17 (PCR) and 0.18 (MCA) for counts at Ice Harbor Dam (Table 3). For counts at Priest Rapids Dam, the scaled RMSEPs were about twice as large, at 0.38 and 0.37. Whether this was due to higher interannual variation (i.e., random noise or observation error) in the smaller stock, a poorer relationship with the indicators, or some combination of these is not known. There were two years in particular (2000 and 2002) with large prediction errors for counts at Priest Rapids Dam. The two response variables representing interior stocks of salmon were correlated with each other through time (evident in Figure 4) and predictions for return year 2012 were within the respective ranges of observed values during the previous 10 years for all three data sets (Figure 4).

**Table 3:** Model performance and predictions (in thousands of fish) for fish returning in 2012.

		Prediction	Prediction Interval	RMSEP	Scaled RMSEP	Fitted R^2^
Bonneville Dam (adjusted for downstream harvest)	PCR	178	118-268	39	0.20	0.86
	MCA	179	126-256	39	0.20	
Ice Harbor Dam	PCR	68	43-110	13	0.17	0.85
	MCA	68	46-102	14	0.18	
Priest Rapids Dam	PCR	14	7-28	6.3	0.38	0.69
	MCA	14	8-26	6.1	0.37	

Root Mean Squared Error of Prediction (RMSEP) is from leave-one-out cross validation, scaled RMSEP = RMSEP/mean observed returns.

**Figure 5:**
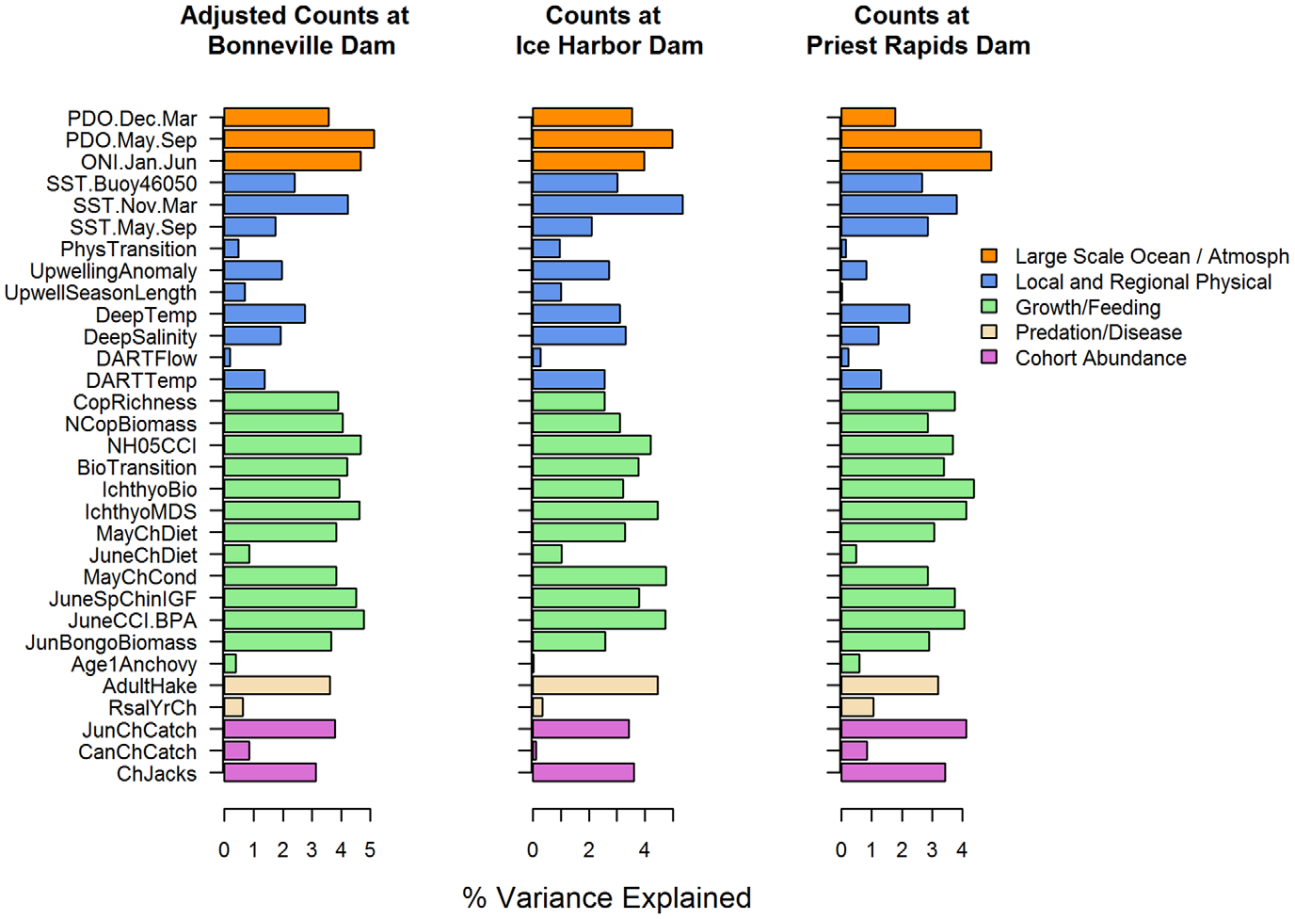
Indicator importance values. Percent of variance in salmon returns explained in the MCA analysis that can be attributed to each indicator (Table 1).

None of the indicators included in the analysis clearly stood out as the best predictor of salmon returns; there was a broad distribution of contributions to model fits from the indicators (Figure 5). Yet, a few significant results emerged from the variable importance values. Among the top contributing indicators to spring Chinook salmon were several measures of potential salmon prey and salmon growth (e.g., JuneCCI.BPA, IchthyoCI, and JuneChIGF) as well as some indices representing large-scale sea surface temperatures (PDO.May.Sep and ONI.Jan.Jun). For all three response variables, indicators in Categories 3 (growth/feeding) and 1 (large-scale ocean and atmospheric) had the highest average importance (Table 4).

**Table 4:** Variable importance by category and response variable.

	Large-scale ocean and atmospheric	Local and regional physical	Growth and feeding	Predation and disease	Cohort abundance
Adjusted counts at Bonneville Dam	4.45	1.77	3.63	2.12	2.59
Counts at Ice Harbor Dam	4.16	2.44	3.19	2.39	2.38
Counts at Priest Rapids Dam	3.79	1.53	3.07	2.18	2.80

Values represent the average weight of all indicators within a category.

In contrast, most indicators in Category 2 (local and regional) played a small role. For each of the three response variables, there was only one local and regional indicator (SST.Nov.Mar) that ranked among the top ten. Interestingly, this particular indicator represented the temperature during the winter prior to ocean entry, suggesting that the relationship between winter ocean temperature and salmon survival is indirect, and perhaps operates mechanistically by mediating ocean productivity or prey resources the following spring, as suggested by Logerwell et al. [39]. Supporting this concept is the lower rank of the sea surface temperature indicator from May through September (Figure 5).

Contributions from measures of cohort abundance (Category 5) were surprisingly mediocre, with the indicator for jack abundance in the year prior to adult return (ChJacks) ranking 11^th^ to 18^th^ out of 31 indicators. Indeed, many of the indicators that had been found significant in other modeling efforts [10], [13], [19] showed little contribution in this analysis. Indicators representing the Columbia River environment ranked between 22^st^ and 24^th^ (DARTTemp) or were near the bottom of the ranking (DARTFlow) in all three MCA analyses. Similarly, the two upwelling indices (UpwellSeasonLength and UpwellingAnomaly) had very low weights in all three analyses.

## Discussion

We found that almost all indicators related to feeding and growth (Category 3) were important in forecasting adult returns to the Columbia River. Although inferential, this information helps fill gaps in our understanding of salmon marine ecology. For many salmon species, we know that larger and faster-growing fish tend to survive better in marine waters [8], [9], but we know less about precisely when this mortality occurs. Both copepods and ichthyoplankton metrics, which are known to contribute to Chinook salmon diets [22], were important here. Furthermore, diet composition was much more important in May than in June (Figure 5), representing the importance of the timing of the onset of piscivory. We also know that abundance of yearling Chinook in our coastal survey conducted in June is more correlated with adult returns than the same survey conducted in May (unpublished data). Moreover, Tomaro et al. [40] showed that size at marine entry was not related to adult returns, but size about one month later was significantly related to returns. Together, these results suggest that feeding, growth, and concomitant mortality between May and June are important drivers for setting salmon year-class strength.

It should be noted, however, that these growth-centric, bottom-up results do not necessarily diminish the importance of top-down drivers of yearling Chinook salmon survival. Choosing between movement and predator avoidance is often a tradeoff [41]. Fish that exhibit a strong northward migration, as these fish do in the marine environment, may be more susceptible to coastal or pelagic predators. Due to data paucity, we included only one predator data set (AdultHake; see [42]), which performed better than average in terms of variable importance, particularly for Upper Columbia River spring Chinook salmon. Inclusion of additional predator data sets, when available, could better inform these results. For example, large numbers of piscivorous seabirds occupy the Columbia River estuary, plume, and coastal environments [43]. These birds consume salmonids and likely affect adult return rates, but time series of bird abundance for this area were too short to be included in the current analysis.

We also found that large-scale oceanic and atmospheric indicators (Category 1) accounted for a large amount of the variability in adult returns. The populations of salmon modeled here quickly migrate north after emigrating from the Columbia River [44]–[46] and experience conditions across a wide spatial extent. Therefore, large-scale patterns of ocean temperature (represented by the PDO and ONI indices) and resulting ecosystem processes have the potential to influence salmon during a vastly longer time period than many of the other indicators, which likely contributed to their high weights in our models (Figure 5). The relationship between PDO and salmon has been explored extensively [15], [47] and some of the biggest changes in marine survival are observed during regime shifts [48], [49]. The last major regime shift in the North Pacific occurred in 1998 [50], which was prior to the data in this analysis. Therefore, as a note of caution, the effect of PDO and other large-scale atmospheric metrics on salmon returns in our model is dependent on being in the current regime and when a new regime is entered, forecasts would benefit from a refitting of the model. Ideally, this would involve a model structure that accommodates regime shifts directly, though in some cases it may be enough to refit the model with data before and after the regime shift. The magnitude and direction of the effect of PDO found here matches qualitatively with what has been shown from analyses straddling a regime shift [24].

Category 2 (local and regional physical) indicators did not fare as well as the large-scale indicators, likely because fish reside in these areas for only a limited time. Therefore, abiotic conditions off of Oregon and Washington are potentially important only for short periods of time or in indirect ways, particularly in their relationship with marine productivity and the prey biomass supported at lower trophic levels. As an example, salmon are known to behaviorally thermoregulate [51], suggesting direct effects of suboptimal temperature can be, to some degree, minimized through behavior. Yet food resources such as larval fish may not have as much behavioral flexibility, allowing temperature to indirectly affect salmon growth and survival through its effect on prey resources. However, it is not possible to capture this fine-scale environmental variance and associated predator and prey behaviors in a regional index. Although growth and mortality are almost certainly related to local conditions, local and regional indicators may be less useful for predictive models than large-scale indicators, at least for the stocks analyzed here. These results support the proposition by Peterman et al. [17] to use only covariates in salmon forecasting models whose correlation extends over geographic areas at least as large as the response variable. In this regard, we hypothesize that other stocks of Chinook salmon or other salmonid species, whose spatial distribution may be more limited [52], will show higher weights for local and regional indicators.

Using the combined information contained in 31 potential indicators of salmon ocean survival, we were able to model spring Chinook salmon adult returns quite well, with a coefficient of determination of 0.86 (from PCR) for spring Chinook salmon returning to the mouth of the Columbia River through 2011. In addition to predicting the 2012 adult return year, it is important to note that predictions for the 2011 return year (created during the leave-one-out procedure) were based solely on data previous to that year, resulting in two true forecasts (i.e., for the 2011 and 2012 adult return years). In 2011, observed adult returns were just over 221 thousand fish, which is almost exactly what the model predicted (the prediction was off by 6 fish; Figure 4). In 2012, observed returns to Bonneville Dam were just over 186 thousand, and a preliminary estimate of harvest downstream of Bonneville Dam was just over 16 thousand fish (Enrique Patino, NOAA Fisheries, unpublished data), suggesting that the final return of adult spring Chinook salmon to the mouth of the Columbia River in 2012 was approximately 203 thousand fish. The predictions for adult returns in 2012 from the current effort was 179 thousand, an error of 11.8%. The accuracy of this model stems, in part, from the inclusion of indicators representing many different aspects of the marine environment. Indeed, models that used a smaller number of ocean indicators suggested that 300 to 600 thousand spring Chinook salmon would return in 2012 (http://www.cbr.washington.edu/crisprt/adult_preseason.html).

Counts at Ice Harbor Dam were underestimated in both 2011 (86 thousand predicted versus 96 thousand observed) and 2012 (68 thousand predicted versus 86 thousand observed), an average error of just over 15%. Counts at Priest Rapids Dam were overestimated in 2011 (17.8 thousand predicted versus 15.2 thousand observed), but underestimated in 2012 (14.4 thousand predicted versus 19.5 thousand observed), an average error of just over 21%. For both populations, these observed returns in 2012 were similar to the average over the last decade (Figure 4).

Most interior Columbia River spring Chinook salmon enter the ocean in May or June and migrate north towards Canada and Alaska [44], [52]. Juvenile fish from the Upper Columbia River spring and the Snake River spring/summer Chinook salmon ESUs have similar marine distributions shortly after ocean entry (David Teel, NOAA Fisheries, unpublished data). This suggests that the marine environment could have a comparable influence on their growth and survival (see [53] for an example of this in sockeye salmon). Indeed, we observed a correlation of 0.81 between the importance of indicators for adult returns from analyses of these two ESUs. However, there were some differences as well. Catches of yearling Chinook salmon during our June coastal salmon survey (JuneChCatch) were better predictors for returns to Priest Rapids Dam (Upper Columbia River spring Chinook salmon) than for returns to Ice Harbor Dam (Snake River spring/summer Chinook salmon). This could be due in part to the timing of our coastal survey relative to juvenile salmon migration or to potentially different marine migration rates between the two ESUs. Similarly, temperatures during the previous winter (SST.Nov.Mar) appeared more important for Snake River fish than Upper Columbia River fish, though the mechanisms for this difference are unclear.

There is an important difference between PCR and MCA that has implications for these results and their use in management. In PCR, the first step is to run a PCA on the indicators, which reduces the dimensions of the indicators without regard to their relationship with the response variable. Consequently, if applied to multiple stocks or species, the PCR loadings for each indicator will be constant across response variables, and the only refinement possible is the inclusion or exclusion of particular PCs. On the other hand, MCA allows the response variable to influence the weighting function (through the covariance matrix). Therefore, application of MCA to multiple stocks or species can result in a fine-tuning of the indicator data to maximize relationships to the appropriate response variable. As an example, SST.Nov.Mar was weighted highest for adult returns to Ice Harbor Dam, which represent the Snake River spring/summer Chinook salmon ESU. However, SST.Nov.Mar was relatively less important for the other two adult return groups (Figure 5). If the goal of management is to summarize the ocean environment in general terms for management of multiple stocks, PCR may be the appropriate choice of methods. However, if the management goal is to make forecasts of individual stocks, MCA provides the flexibility to weight the indicators specifically for that stock.

The modeling approach demonstrated here promises to be important to salmon management in the Pacific Northwest. Many current forecasting models rely on one or two indices to predict returns for the following year. Yet, ocean survival is the result of complex interactions among the physical environment and organisms at multiple trophic levels; thus ocean survival is driven by temporal and spatial dynamics that cannot be summarized by just a couple indices of the physical environment. By combining a large number of indicators, particularly ones with a direct link to growth or survival such as predator or prey resources, this approach avoids the pitfalls of relying too heavily on any one indicator.

We made several attempts to simplify the set of indicators through model selection techniques. However, we strongly recommend against this practice when using a large number of indicators. As an example of the danger of *post hoc* indicator selection, we ran a leave-one-out (LOO) analysis on the indicators (sequentially removed each indicator and ran the full model, keeping track of the improvement in RMSEP). After removing the indicator whose absence made the most improvement in model fit, we ran the LOO procedure again. This process was continued until no further reduction of the RMSEP could be obtained. In a simple linear model, this process would be comparable to a backwards selection of predictor variables. Yet when using PCR and MCA, this process lead to combinations of indicators with spurious relationships to the response variable. To convince ourselves of this, we randomized the indicator data (within each indicator, among years) and ran the above analysis. Using these 31 randomized variables, the resulting model correlated with observed salmon counts with an R^2^ of greater than 0.9. We therefore suggest all indicator selection be done *a priori* when using these multivariate methods.

Finally, the expectation of future data collection can play a critical role. Many of the indicators in this analysis were obtained at great cost (in both time and money), while others can be obtained remotely via satellites or from various websites (PDO, ONI, upwelling, river flow). Therefore, the decision of whether or not to include a particular indicator depends on the goal of the research and expected future applications of the model. However, restricting analyses to just those indicators likely to exist in the future can greatly influence model forecasts. As an example, we ran the MCA analysis on a simplified set of 9 indicators that will almost certainly be available for many years (PDO.Dec.Mar, PDO.May.Sep, ONI.Jan.Jun, SST.Buoy46050, UpwellingAnomaly, UpwellSeasonLength, DARTTemp, DARTFlow, and ChJacks). Compared to the full set of 31 indicators, the RMSEP (average error in predictions) almost doubled. In addition, prediction intervals were larger by about 25%, suggesting that the less certain (and costlier) indicators significantly improve forecasts. That stated, the current list in our analysis is by no means definitive, nor is it comprehensive (e.g., there is a distinct lack of salmon predator indicators). Future efforts will focus on techniques to refine the set of included indicators. We also note that using measures of marine survival directly would be a more appropriate response variable than using counts of returning adults. However, survival estimates require both smolt abundance and adult age structure data, which do not exist for many of these populations. As these data become available, model fits and forecasting ability will likely improve.

Each year, fisheries management agencies set a fishing quota for each stock of Pacific salmon in the Columbia River, which is then divided among recreational, commercial, and tribal fishers. Not only is this a multi-million dollar fishery, but most of the stocks in this analysis are listed under the Endangered Species Act as either threatened or endangered [2]. Therefore, the cost of inaccurately predicting returns, to fish and fishers, is significant. By optimizing the available information to estimate the number of fish that will return one to two years in the future, managers can more efficiently apportion catch and plan for future scenarios, resulting in more equitable fisheries and a better chance of recovering these threatened and endangered species.

## References

[1] Weitkamp LA, Bentley PJ, Litz MNC (2012) Seasonal and interannual variation in juvenile salmonids and associated fish assemblage in open waters of the lower Columbia River estuary, U.S.A. Fishery Bulletin.

[2] Ford MJ, Cooney T, McElhany P, Sands N, Weitkamp L, et al. (2010) Status review update for Pacific salmon and steelhead listed under the Endangered Species Act.

[3] Quinn TP (2005) The Behavior And Ecology Of Pacific Salmon And Trout: American Fisheries Society.

[4] Beamish RJ, Mahnken C (2001) A critical size and period hypothesis to explain natural regulation of salmon abundance and the linkage to climate and climate change. Progress In Oceanography 49: 423–437.

[5] Duffy EJ (2009) Factors during early marine life that affect smolt-to-adult survival of ocean-type Puget Sound Chinook salmon (*Oncorhynchus tshawytscha*). Seattle, WA: University of Washington.

[6] Holtby LB, Andersen BC, Kadowaki RK (1990) Importance of Smolt Size and Early Ocean Growth to Interannual Variability in Marine Survival of Coho Salmon (*Oncorhynchus kisutch*). Canadian Journal of Fisheries and Aquatic Sciences 47: 2181–2194.

[7] Wells BK, Grimes CB, Sneva JG, McPherson S, Waldvogel JB (2008) Relationships between oceanic conditions and growth of Chinook salmon (*Oncorhynchus tshawytscha*) from California, Washington, and Alaska, USA. Fisheries Oceanography 17: 101–125.

[8] Moss JH, Beauchamp DA, Cross AD, Myers KW, Farley EV, et al (2005) Evidence for Size-Selective Mortality after the First Summer of Ocean Growth by Pink Salmon. Transactions of the American Fisheries Society 134: 1313–1322.

[9] Cross A, Beauchamp D, Moss J, Myers K (2009) Interannual Variability in Early Marine Growth, Size-Selective Mortality, and Marine Survival for Prince William Sound Pink Salmon. Marine and Coastal Fisheries: Dynamics, Management, and Ecosystem Science 1: 57–70.

[10] ODFW WDFW (2012) 2012 Joint Staff Report: Stock Status and Fisheries for Spring Chinook, Summer Chinook, Sockeye, Steelhead, and Other Species, and Miscellaneous Regulations.

[11] Nelson GC (2005) Drivers of ecosystem change. Ecosystems and human well-being Current state and trends findings of the Condition and Trends Working Group of the Millennium Ecosystem Assessment. Washington, DC: Island Press.

[12] Burnham KP, Anderson DR (2010) Model Selection and Multi-Model Inference: A Practical Information-Theoretic Approach: Springer.

[13] Peterman RM (1982) Model of Salmon Age Structure and Its Use in Preseason Forecasting and Studies of Marine Survival. Canadian Journal of Fisheries and Aquatic Sciences 39: 1444–1452.

[14] Geiger HJ, Frenette B, Hart D (1997) Run Forecasts and Harvest Projections for 1997 Alaska Salmon Fisheries and Review of the 1996 Season. Alaska Department of Fish and Game.

[15] Rupp DE, Wainwright TC, Lawson PW, Peterson WT (2012) Marine environment-based forecasting of coho salmon (*Oncorhynchus kisutch*) adult recruitment. Fisheries Oceanography 21: 1–19.

[16] Wang S, Morishima G, Sharma R, Gilbertson L (2009) The Use of Generalized Additive Models for Forecasting the Abundance of Queets River Coho Salmon. North American Journal of Fisheries Management 29: 423–433.

[17] Peterman RM, Pyper BJ, Mueter FJ, Haeseker SL, Su Z, et al. (2009) Statistical Models of Pacific Salmon that Include Environmental Variables. American Fisheries Society Symposium: 125–146.

[18] Noakes DJ, Welch DW, Henderson M, Mansfield E (1990) A Comparison of Preseason Forecasting Methods for Returns of Two British Columbia Sockeye Salmon Stocks. North American Journal of Fisheries Management 10: 46–57.

[19] Scheuerell MD, Williams JG (2005) Forecasting climate-induced changes in the survival of Snake River spring/summer Chinook salmon (*Oncorhynchus tshawytscha*). Fisheries Oceanography 14: 448–457.

[20] Zhou S (2003) Application of Artificial Neural Networks for Forecasting Salmon Escapement. North American Journal of Fisheries Management 23: 48–59.

[21] Haeseker SL, Peterman RM, Su Z, Wood CC (2008) Retrospective Evaluation of Preseason Forecasting Models for Sockeye and Chum Salmon. North American Journal of Fisheries Management 28: 12–29.

[22] Daly E, Brodeur R, Weitkamp L (2009) Ontogenetic Shifts in Diets of Juvenile and Subadult Coho and Chinook Salmon in Coastal Marine Waters: Important for Marine Survival? Transactions of the American Fisheries Society 138: 1420–1438.

[23] Bi H, Peterson WT, Lamb J, Casillas E (2011) Copepods and salmon: characterizing the spatial distribution of juvenile salmon along the Washington and Oregon coast, USA. Fisheries Oceanography 20: 125–138.

[24] Peterson WT, Morgan CA, Peterson JO, Fisher JL, Burke BJ, et al. (2012) Ocean Ecosystem Indicators of Salmon Marine Survival in the Northern California Current. NOAA Fisheries.

[25] Emmett RL, Krutzikowsky GK, Bentley P (2006) Abundance and distribution of pelagic piscivorous fishes in the Columbia River plume during spring/early summer 1998–2003: Relationship to oceanographic conditions, forage fishes, and juvenile salmonids. Progress In Oceanography 68: 1–26.

[26] Mazur MM, Beauchamp DA (2006) Linking piscivory to spatial-temporal distributions of pelagic prey fishes with a visual foraging model. Journal of Fish Biology 69: 151–175.

[27] Brodeur RD, Daly EA, Schabetsberger RA, Mier KL (2007) Interannual and interdecadal variability in juvenile coho salmon (*Oncorhynchus kisutch*) diets in relation to environmental changes in the northern California Current. Fisheries Oceanography 16: 395–408.

[28] Martin F, Hedger RD, Dodson JJ, Fernandes L, Hatin D, et al (2009) Behavioural transition during the estuarine migration of wild Atlantic salmon (*Salmo salar* L.) smolt. Ecology of Freshwater Fish 18: 406–417.

[29] Biro PA, Post JR, Abrahams MV (2005) Ontogeny of energy allocation reveals selective pressure promoting risk-taking behaviour in young fish cohorts. Proceedings of the Royal Society B: Biological Sciences 272: 1443–1448. 16011918 10.1098/rspb.2005.3096 PMC1559824

[30] Anderson JJ, Beer WN (2009) Oceanic, riverine, and genetic influences on spring chinook salmon migration timing. Ecological Applications 19: 1989–2003. 20014573 10.1890/08-0477.1

[31] Hyun S-Y, Hilborn R, Anderson JJ, Ernst B (2005) A statistical model for in-season forecasts of sockeye salmon (*Oncorhynchus nerka*) returns to the Bristol Bay districts of Alaska. Canadian Journal of Fisheries and Aquatic Sciences 62: 1665–1680.

[32] Peterson WT, Morgan CA, Fisher JP, Casillas E (2010) Ocean distribution and habitat associations of yearling coho (*Oncorhynchus kisutch*) and Chinook (*O. tshawytscha*) salmon in the northern California Current. Fisheries Oceanography 19: 508–525.

[33] Brodeur RD, Fisher JP, Emmett RL, Morgan CA, Casillas E (2005) Species composition and community structure of pelagic nekton off Oregon and Washington under variable oceanographic conditions. Marine Ecology Progress Series 298: 41–57.

[34] Legendre P, Legendre L (1998) Numerical ecology: Elsevier.

[35] Koslow JA, Hobday AJ, Boehlert GW (2002) Climate variability and marine survival of coho salmon (*Oncorhynchus kisutch*) in the Oregon production area. Fisheries Oceanography 11: 65–77.

[36] Jolliffe IT (2002) Principal component analysis: Springer-Verlag.

[37] Esbensen KH, Guyot D, Westad F, Houmøller LP (2002) Multivariate Data Analysis - in Practice: An Introduction to Multivariate Data Analysis and Experimental Design: Camo Process AS.

[38] Peres-Neto PR, Jackson DA, Somers KM (2003) Giving meaningful interpretation to ordination axes: Assessing loading significance in principal component analysis. Ecology 84: 2347–2363.

[39] Logerwell EA, Mantua N, Lawson PW, Francis RC, Agostini VN (2003) Tracking environmental processes in the coastal zone for understanding and predicting Oregon coho (*Oncorhynchus kisutch*) marine survival. Fisheries Oceanography 12: 554–568.

[40] Tomaro LM, Teel DJ, Peterson WT, Miller JA (2012) When is bigger better? Early marine residence of middle and upper Columbia River spring Chinook salmon. Marine Ecology Progress Series 452: 237–252.

[41] Sih A (1987) Predators and preylifestyles: an evolutionary and ecological overview. *In*: Kerfoot WC, Sih A, editors. Predation: Direct and Indirect Impacts on Aquatic Communities. Hanover: University Press of New England. 203–224.

[42] Emmett R, Krutzikowsky G (2008) Nocturnal Feeding of Pacific Hake and Jack Mackerel off the Mouth of the Columbia River, 1998–2004: Implications for Juvenile Salmon Predation. Transactions of the American Fisheries Society 137: 657–676.

[43] Schreck CB, Stahl TP, Davis LE, Roby DD, Clemens BJ (2006) Mortality estimates of juvenile spring-summer Chinook salmon in the Lower Columbia River and estuary, 190–1998: Evidence for delayed mortality? Transactions of the American Fisheries Society 135: 457–475.

[44] Trudel M, Fisher J, Orsi JA, Morris JFT, Thiess ME, et al (2009) Distribution and migration of juvenile Chinook salmon derived from coded wire tag recoveries along the continental shelf of western North America. Transactions of the American Fisheries Society 138: 1369–1391.

[45] Weitkamp L (2010) Marine distributions of Chinook salmon from the west coast of North America determined by coded wire tag recoveries. Transactions of the American Fisheries Society 139: 147–170.

[46] Daly EA, Brodeur RD, Fisher JP, Weitkamp LA, Teel DJ, et al (2012) Spatial and trophic overlap of marked and unmarked Columbia River Basin spring Chinook salmon during early marine residence with implications for competition between hatchery and naturally produced fish. Environmental Biology of Fishes 94: 117–134.

[47] Wells BK, Grimes CB, Field JC, Reiss CS (2006) Covariation between the average lengths of mature coho (*Oncorhynchus kisutch*) and Chinook salmon (*O. tshawytscha*) and the ocean environment. Fisheries Oceanography 15: 67–79.

[48] Hare SR, Francis RC (2004) Climate change and salmon production in the Northeast Pacific Ocean. In: Beamish RJ, editor. Climate Change and Northern Fish Populations: Can. Spec. Publ. Fish. Aquat. Sci.

[49] Pearcy WG (1992) Ocean ecology of North Pacific salmonids: Washington Sea Grant Program.

[50] Peterson WT, Schwing FB (2003) A new climate regime in northeast pacific ecosystems. Geophysical Research Letters 30.

[51] Hinke JT, Foley DG, Wilson C, Watters GM (2005) Persistent habitat use by Chinook salmon Oncorhynchus tshawytscha in the coastal ocean. Marine Ecology Progress Series 304: 207–220.

[52] Tucker S, Trudel M, Welch DW, Candy JR, Morris JFT, et al (2011) Life history and seasonal stock-specific ocean migration of juvenile Chinook salmon. Transactions of the American Fisheries Society 140: 1101–1119.

[53] McKinnell S, Reichardt M (2012) Early marine growth of juvenile Fraser River sockeye salmon (*Oncorhynchus nerka*) in relation to juvenile pink (*Oncorhynchus gorbuscha*) and sockeye salmon abundance. Canadian Journal of Fisheries and Aquatic Sciences 69: 1499–1512.

[54] Bograd SJ, Schroeder I, Sarkar N, Qiu X, Sydeman WJ, et al. (2009) Phenology of coastal upwelling in the California Current. Geophysical Research Letters 36.

[55] Hooff RC, Peterson WT (2006) Copepod Biodiversity as an Indicator of Changes in Ocean and Climate Conditions of the Northern California Current Ecosystem. Limnology and Oceanography 51: 2607–2620.

[56] Keister JE, Di Lorenzo E, Morgan CA, Combes V, Peterson WT (2011) Zooplankton species composition is linked to ocean transport in the Northern California Current. Global Change Biology 17: 2498–2511.

[57] Litz MNC (2008) Ecology of the Northern Subpopulation of Northern Anchovy (*Engraulis mordax*) in the California Current Large Marine Ecosystem: Oregon State University.

